# Solid Lipid Nanoparticles, an Alternative for the Treatment of Triple-Negative Breast Cancer

**DOI:** 10.3390/ijms251910712

**Published:** 2024-10-05

**Authors:** Monserrat Llaguno-Munive, Maria Ines Vazquez-Lopez, Patricia Garcia-Lopez

**Affiliations:** 1 Laboratorio de Física Médica, Subdirección de Investigación Básica, Instituto Nacional de Cancerología, Mexico City 14080, Mexico; muniv1250@hotmail.com; 2 Laboratorio de Fármaco-Oncología y Nanomedicina, Subdirección de Investigación Básica, Instituto Nacional de Cancerología, Mexico City 14080, Mexico; manevazlo@gmail.com

**Keywords:** solid lipid nanoparticles, breast cancer, triple-negative breast cancer, nanomedicine

## Abstract

Within the field of nanomedicine, which is revolutionizing cancer treatment, solid lipid nanoparticles (SLNs) have shown advantages over conventional chemotherapy when tested on cancer cells in preclinical studies. SLNs have proven to be an innovative strategy for the treatment of triple-negative breast cancer cells, providing greater efficiency than existing treatments in various studies. The encapsulation of antineoplastic drugs in SLNs has facilitated a sustained, controlled, and targeted release, which enhances therapeutic efficiency and reduces adverse effects. Moreover, the surface of SLNs can be modified to increase efficiency. For instance, the coating of these particles with polyethylene glycol (PEG) decreases their opsonization, resulting in a longer life in the circulatory system. The creation of positively charged cationic SLNs (cSLNs), achieved by the utilization of surfactants or ionic lipids with positively charged structural groups, increases their affinity for cell membranes and plasma proteins. Hyaluronic acid has been added to SLNs so that the distinct pH of tumor cells would stimulate the release of the drug and/or genetic material. The current review summarizes the recent research on SLNs, focusing on the encapsulation and transport of therapeutic agents with a cytotoxic effect on triple-negative breast cancer.

## 1. Introduction

Breast cancer continues to be a challenge for health services around the world. Despite advances in prevention, diagnosis, and treatment, the incidence and mortality rates of this disease continue to rise from year to year. Breast cancer is the most frequently reported malignant neoplasm worldwide. The 2.3 million new cases of breast cancer diagnosed around the world in 2020 constituted 11.7% of new cases of all cancers, representing a higher rate of incidence than that found for lung, colon, or prostate cancer [1], according to data provided by the International Agency for Research on Cancer (IARC, 2020).

Two major difficulties diminish the effectiveness of breast cancer treatment. Firstly, the diagnosis is often made in the advanced stages of the disease, at which time the patients are undergoing metastasis. Under this condition, there are limited therapeutic options capable of eliminating cancer cells. Secondly, cancer cells commonly acquire resistance mechanisms to chemotherapy drugs, causing some patients who initially respond well to chemotherapy to become insensitive to treatment [2].

According to the problems with conventional treatments for TNBC, it is necessary to continue researching new treatments. The purpose of the current review is to summarize the general information about TNBC, which is essential to understanding the characteristics of this subtype of breast cancer that does not express hormone receptors typically targeted for standard treatments, complicating its current treatment. This requires the exploration of innovative strategies, such as SLNs. So, the progress in the development of SLNs and the most recent advances in the encapsulation and transport of therapeutic agents and nucleic acids are also discussed in the current contribution by reviewing relevant reports on the potential role of SLNs for TNBC treatment.

## 2. Classification of Breast Cancer

**Figure 1:**
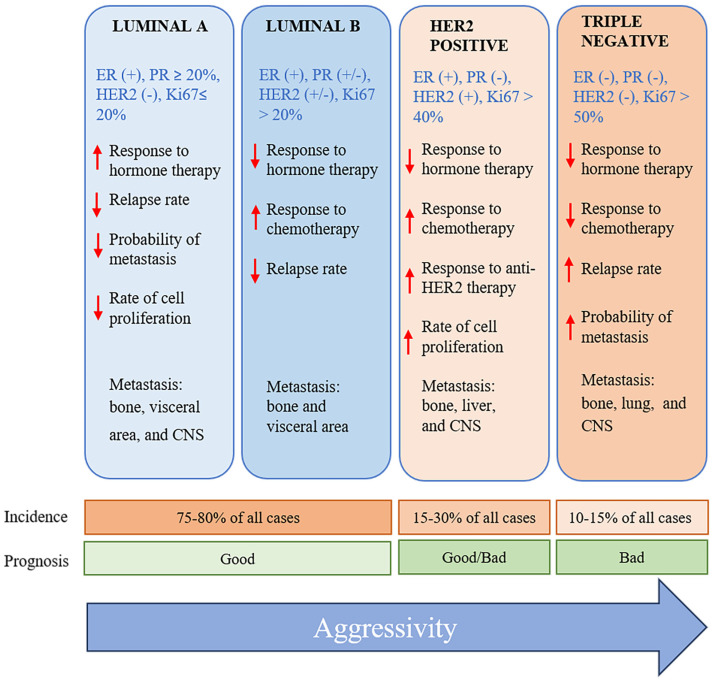
Molecular classification of breast cancer, listing the principal characteristics of each subtype [6]. ER, estrogen receptor; PR, progesterone receptor; HER2, human epidermal growth factor 2. The red down-pointing or up-pointing arrows indicate a decrease or increase, respectively. The blue arrow to the right indicates an increase in aggressiveness.

Breast cancer has been classified by using diverse criteria, including histology, molecular characteristics, and the expression of specific biomarkers. The establishment of the specific characteristics of breast cancer tumors allows for the selection of the most effective treatment. Regarding the molecular perspective, among the various subtypes of breast cancer are luminal A, luminal B, normal-like, HER2-enriched, basal-like, and triple-negative [3,4] (Figure 1). The triple-negative subtype comprises 10–20% of all cases of breast cancer. It is found at a greater prevalence in Afro-descendants, women under 40 years of age, and/or those with a mutation in the BCRA1 gene [5].

## 3. Triple-Negative Breast Cancer: Characteristics and New Perspectives for Treatment

Triple-negative breast cancer (TNBC) is characterized by the absence or low expression of estrogen receptors (ERs), progesterone receptors (PRs), and human epidermal growth factor 2 (HER2) receptors. Since these receptors are the targets of many chemotherapy drugs, their limited expression makes TNBC the breast tumor type with the least number of therapeutic options available [7].

**Figure 2:**
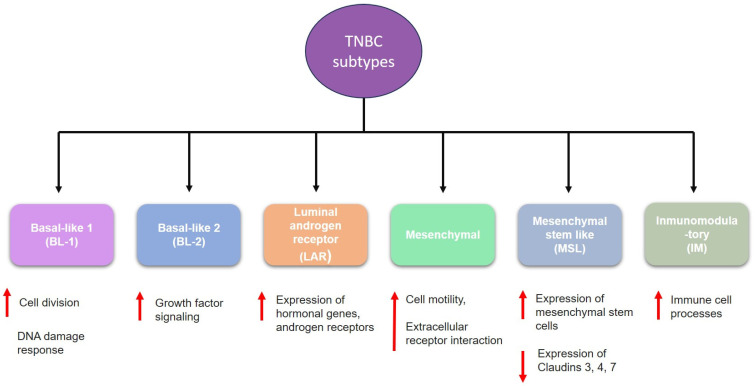
Classifications of triple-negative breast cancer (TNBC). The red down-pointing or up-pointing arrows indicate a decrease or increase, respectively.

TNBC is highly heterogeneous and classified into six subtypes (Figure 2); these subtypes have different molecular and biological characteristics that could influence prognosis and treatment response. These subtypes are as follows:

(1) **Basal-like 1 (BL-1)** is characterized by increased cell division and elevated DNA damage response pathways (ATR/BCA) [8,9].
(2) **Basal-like 2 (BL-2)** involves increased growth factor signaling pathways such as epidermal growth factor (EGF), nerve growth factor (NGF), and Wnt/β-catenin [8,10].
(3) **Luminal androgen receptor (LAR)** is characterized by high levels of androgen receptor genes and alterations of PI3K pathway genes [8,11,12].
(4) **Mesenchymal (M**) has elevated expression of genes involved in epithelial–mesenchymal transition (EMT), and it is enriched in components involved in cell motility [8,13].
(5) **Mesenchymal stem-like (MLS)** shares the overexpression of genes related to EMT; however, it is also characterized by enrichment in genes involved in angiogenesis, including VEGFR2 and high expression of stem cell genes [8,14].
(6) **Immunomodulatory (IM)** is enriched within genes that regulate immune response and antigen processing [8].

Chemotherapy with cytotoxic agents affords the greatest rate of success for TNBC patients, above all if administered in the early stages of the disease. Some of the most common drugs for TNBC are anthracyclines (doxorubicin), taxanes (paclitaxel), and alkylating agents (cyclophosphamide). Treatment in the advanced stages involves antimetabolites (gemcitabine and capecitabine), inhibitors of microtubules (eribulin), and platinum derivatives (carboplatin) [7]. Although the initial response to chemotherapy is good in patients with TNBC, 90% of cases acquire resistance to pharmacological treatment. Indeed, drug resistance in tumors is the main cause of therapeutic failure [2].

Nanomedicine is an alternative to conventional treatments and is one of the areas of science presently showing the most rapid growth. It involves the integration of several branches of research, including medicine, pharmacology, molecular biology, and gene therapy. In recent years, nanoparticles have been developed as systems for the transport and release of drugs in order to achieve greater efficiency in the treatment of diverse diseases (e.g., cancer). In various preclinical studies, nanoparticles have proven to be advantageous as a form of encapsulation and transport, being capable of selectively delivering drugs to target receptors. Such studies carried out during the last few years have generally shown a better pharmacological response to treatment with new therapeutic strategies based on nanotechnology than with conventional treatments.

Regarding TNBC, the recent development of nanoparticle systems gives rise to new therapeutic alternatives with the potential of being more efficacious and personalized. Current investigation in this field is focused on optimizing this potential to improve the quality of life of TNBC patients.

## 4. Nanoparticles: Advances and Potential Therapeutic Uses

Nanoparticles have attracted the attention of scientists due to their capacity for diagnosis, medical imaging, and cancer treatment in a single system. They are defined as particles with a diameter of 1–1000 nanometers (nm) [15]. Due to this size range, NSLs can be used in several applications, including cancer therapy, wound regeneration, and cosmetics [16,17,18]. However, a size range of 10–100 nm is considered most suitable for cancer therapy because it can penetrate tumor tissue more quickly due to the enhanced permeability and retention (EPR) effect, which is crucial for effective drug delivery by allowing more significant intratumoral accumulation. Additionally, it has been reported that this size range improves the pharmacokinetic profile, prolongs blood circulation, and minimizes side effects. The small size of nanoparticles enables them to navigate biological barriers, such as cell membranes and extracellular matrices, which is essential for efficient drug delivery. However, nanoparticles with a diameter of less than 10 nm tend to be filtered through the kidneys, increasing their elimination and resulting in minimal intratumoral accumulation [19,20]. On the other hand, those with a diameter greater than 100 nm have a higher probability of being cleared by phagocytosis, reducing their intratumoral accumulation [21].

**Figure 3:**
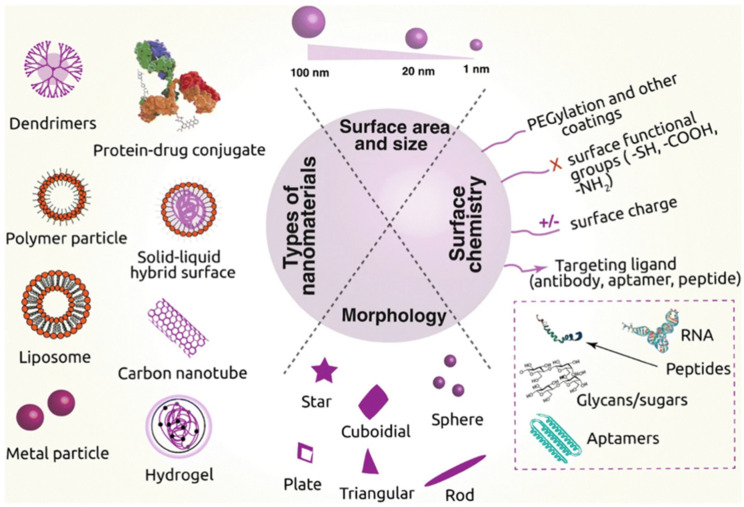
Scheme of the different types of nanoparticles and their main characteristics, including size, shape, coating, surface chemistry, and target. Image from Navya et al. [24].

Also affecting biodistribution is the shape of a nanoparticle, which can be cubical, spherical, rod-shaped, disc-shaped, or hexagonal [22,23,24]. Spherical nanoparticles are reported to be eliminated from the organism more slowly [23]. Additionally, the coating of these particles with different materials (e.g., polyethylene glycol (PEG)) reduces their opsonization, leading to a longer life in the circulatory system and thus an increase in their bioavailability (Figure 3) [24].

Recent studies have demonstrated that nanoparticles have great potential as drug delivery systems due to their capacity to encapsulate hydrophilic as well as hydrophobic compounds. This encapsulation improves bioavailability and protects against biological, physical, and chemical degradation. Moreover, nanoparticles can be directed at a particular organ and because of their size are able to cross biological barriers. They can transport a wide variety of molecules and provide a controlled and targeted release. Compared to conventional therapies, nanosystems are more stable, less toxic to the organism, and more selective for cancer cells [25].

## 5. Structure and Characteristics of Solid Lipid Nanoparticles (SLNs)

Distinct types of nanoparticles are continually being developed to enhance the efficiency of current treatments for TNBC. Within this context, solid lipid nanoparticles (SLNs) are a new generation of nanocarriers with great promise for therapy. Their structure, which is a matrix of solid lipids, has good efficiency of encapsulation and stability as well as low toxicity according to various studies [26,27,28].

**Figure 4:**
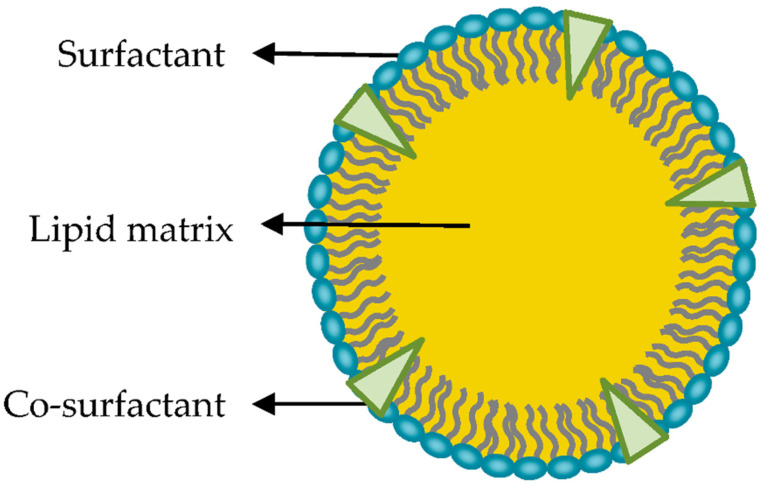
Schematic illustration of the structure of sold lipid nanoparticles (SLNs). Image from Bayon et al. [29].

SLNs are colloidal particles of submicron size, from 50 to 1000 nm in diameter. They consist of a matrix of crystalline lipids that are solid at ambient temperature and some tensoactive agents that allow them to accommodate molecules between their chains of fatty acids, either in the lipid nucleus or on the surface (Figure 4). They may be spherical, ellipsoidal, or disc-like. The surface charge of an SLN is determined by its constituents and the pH of the surrounding medium [27,29,30].

The quality of the formulations of SLNs depends on an adequate proportion of the lipid components. These components should be selected based on the nature of the drug to be incorporated. The drug must be solubilized in the lipid matrix in order to have good efficiency of encapsulation. The substances most commonly used to form the lipid nucleus are mono-, di-, and triglycerides, fatty acids, fatty alcohols, and waxes, which are all biocompatible [29]. The lipid components of the solid matrix are in solid form at room temperature and body temperature. Consequently, they are safe and viable for low-cost mass production. The majority are super-purified waxes and complex emulsions of glycerides [31].

**Figure 5:**
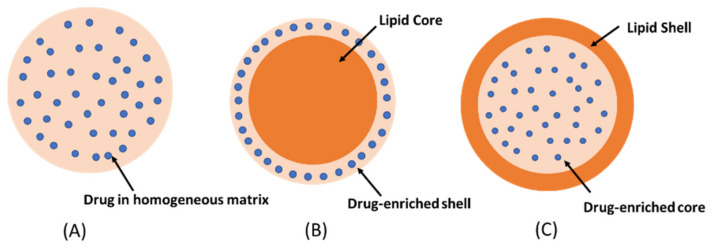
Types of solid lipid nanoparticles (SLNs). (**A**) Drug into homogeneous matrix, (**B**) drug-enriched shell, (**C**) drug-enriched core. Image from Chutoprapat et al. [34].

The formation of a solid matrix is essential because it determines the controlled release of the drug. Moreover, the mobility of a drug in a solid lipid matrix is substantially less than that found in other components. As a system of transport of different active agents, SLNs offer various advantages compared to conventional systems of administration (Table 1) [28,29,32]. The unique properties of SLNs, contingent on their extremely small size and their capacity to incorporate drugs, provide the opportunity to create new therapeutic approaches involving a targeted and controlled release of drugs [33]. Depending on the incorporation of the drug into an SNL, it may be classified into three models: (Figure 5A) drug into the homogeneous matrix, which is formed when the drugs are homogenously dispersed, (Figure 5B) drug-enriched shell, which is characterized by the drug enclosed by an outer shell, and (Figure 5C) drug-enriched core, which is obtained when the drugs crystalize before the lipid. (Figure 5) [34,35].

It has been known that SLNs are stable in aqueous dispersion. To obtain adequate structure and stability, the method of preparation of SLNs is crucial. A transfer of energy to the SLN system is required to generate very small and stable particles in aqueous environments. This energy can originate from distinct sources exhaustively described in the literature: high-pressure or high-speed homogenization, cold or hot homogenization, sonication, and emulsification [27].

Also, it has been reported that SLNs have distinct physical and chemical properties compared to other similar nanosystems, such as reverse micelles and liposomes. Physically, SLNs comprise biodegradable solid lipids, providing a stable matrix that enhances drug loading capacity and bioavailability. SLNs are characterized by their ability to incorporate both hydrophilic and lipophilic drugs, in contrast to micelles, which primarily solubilize hydrophobic drugs. Additionally, SLNs offer sustained drug release due to the use of solid lipids [36,37,38,39]. Overall, SLNs present unique advantages in drug delivery systems, particularly in terms of biocompatibility and stability, which distinguish them from other nanosystems.

In the case of scale, SLNs are cost-effective in terms of raw materials and production, offer outstanding physicochemical stability, and can be sterilized and lyophilized affordably. These features make SLNs well suited for large-scale industrial manufacturing. Shegokar et al. developed batches of stavudine-SLN at lab scale (40 g) to medium scale (10 kg) and large scale (20–60 kg), and all batches were stable. They concluded that homogenization scale-up was relatively easy compared to other processes [40,41]. Compared to metal nanoparticles that use biological synthesis, it has been seen that their scale-up is low cost; however, they present variability in the size and shape of the produced NPs due to differences in the plant species used in their synthesis [42]. In the case of liposomes, although several formulations are already on the market, not all laboratory-scale techniques are easy to scale up for the industrial production of liposomes. Many conventional methods use organic solvents, and residual organic solvents can become toxic, posing a potential risk to human health [43].

While SLNs offer several promising drug delivery features, they also present disadvantages, or limitations, such as the lower drug loading capacity compared to other nanoparticles (polymeric nanoparticles or liposomes), because due to their perfect crystalline structure, during synthesis, they can restrict the amount of drug that can be incorporated, which leads to low encapsulation efficiency [44]. Another drawback is the potential for drug leakage; over time, the encapsulated drug may diffuse out of the SLNs before reaching the target tissue [32], causing a rapid systemic elimination of the released drug. In addition, healthy tissues can be exposed to the toxicity of the free drug.

Although preclinical studies on SLNs have shown a good biocompatibility and safety profile, due to controlled release kinetics of the encapsulated drug, in both in vitro and in vivo models [45] there are often insufficient studies to understand the long-term effects of prolonged usage of an SNL and its chemical components. Strong investigations are needed to evaluate the possible long-term toxicity of SNLs.

### Nanotoxicological Classification System

**Figure 6:**
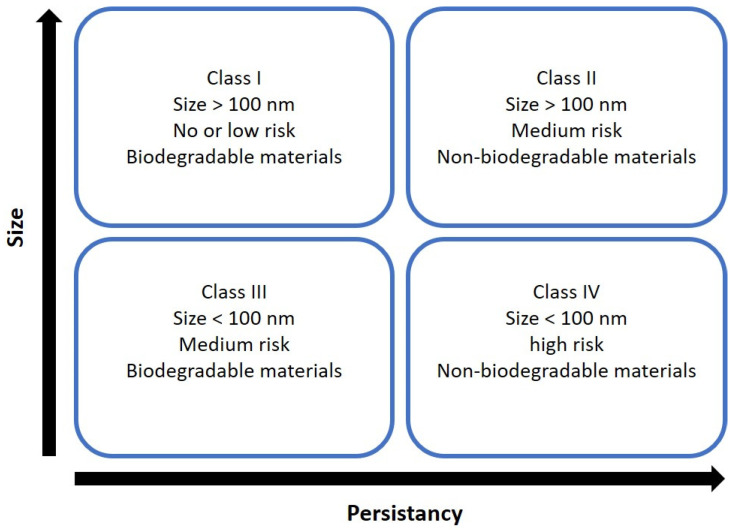
The nanotoxicological classification system (NCS) depends on size and persistency in the body. Image adapted from Keck and Müller [46].

Nanosystems are placed in four categories according to size and biodegradability (Figure 6). SLNs are composed of lipids and natural substances in the body and are considered biodegradable. Therefore, SLNs are typically classified as members of classes I or III [27]. Class I refers to nanosystems composed of biodegradable materials with a size above 100 nm. Since SLNs are made from physiologically compatible lipids such as fatty acids, glycerides, and sterols. Class III, conversely, is composed of biodegradable lipids, but their size is above 100 nm [46].

It has been reported that SLNs remain stable in aqueous dispersion, in vitro studies have shown that SLNs are acceptable at <1 mg/mL concentration of lipids, and nanosystems with high quantities of surfactants have more risk of cytotoxicity [46,47]; however, it is necessary that there are more nanotoxicology studies to understand the effects of SLNs on the body (Figure 6).

## 6. Delivery Mechanism of SLNs

It has been reported that lipid composition, pH, temperature, and the site where the drug is encapsulated influence the drug delivery mechanisms, either through passive or active accumulation.

### 6.1. Passive Delivery

In passive delivery, the properties of SLNs, such as size, composition lipid, and surface characteristics, play a crucial role. One of the main characteristics of tumors is the stimulation of angiogenesis to promote increased nutrients and oxygen. However, blood vessel formation results in a defective architecture with fenestrations (from 200 to 1200 nm) [27,48]. This pathology is the enhanced permeability and retention effect (EPR). Therefore, SLNs accumulate in the tumor through passive diffusion via the EPR effect, penetrating through the fenestrations and remaining there due to poor lymphatic drainage nanoparticles, with sizes ranging from 50 to 200 nm, which can reach passively the tumor site. Surface modification of SLNs with hydrophilic polymers such as polyethylene glycol (PEG) can enhance their circulation time by reducing opsonization and clearance by the reticuloendothelial system (RES), thus promoting passive accumulation at the target site. PEGylation is the most important surface modification of nanoparticles. PEG increases time circulation, and it has been reported that it enhances particle permeation in tumors [49].

### 6.2. Active Delivery

There have been several studies on active delivery; generally, nanoparticles are functionalized by adjusting the composition of SLNs; they can be readily functionalized with various targeting methods to facilitate active delivery against specific cell types of interest. This modification aims to enhance the targeting efficiency of nanoparticles and reduce the side effects [50]. Active delivery involves modifying the surface of nanoparticles with several ligands that are overexpressed on the tumor cells, such as small molecules, peptides, antibodies, polysaccharides, hormones, and nucleic acids. These molecules are recognized by cell receptors or transporters [51,52]. The specific interaction between the surface ligands of nanoparticles and their receptor expression on the target cells could facilitate the internalization process through endocytosis. It has been reported that nanoparticles modified with folate for active targeting showed enhanced cellular uptake by endocytosis [53,54].

### 6.3. Triggered Release

**Figure 7:**
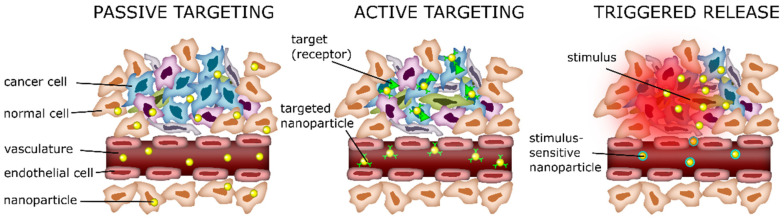
Schematic representation of drug targeting via passive (EPR effect), active, and triggered release. Image from Hafeez et al. [55].

Triggered release mechanisms play a crucial role in drug delivery systems. These drug release mechanisms depend on various stimuli in the tumor microenvironment or external agents to control the release (Figure 7) [55]. Some of the critical stimuli and their associated responses include pH; it is known that tumors often have a lower pH compared to healthy tissues, and SLNs can be designed to respond to this acidic pH to release therapeutic agents. Other stimuli are magnetic field, ultrasonic, and temperature. Magnetic solid lipid nanoparticles use an external magnetic field to control their location, thus avoiding the disadvantages of systemic administration. Once magnetic drug carriers accumulate within a tumor, an externally applied alternating magnetic field (AMF) can increase the drug release and temperature [56]. In the case of ultrasound, waves can trigger the release of drugs from nanoparticles in the tumor area without affecting healthy tissue. It has been reported that exposure to ultrasound produces a reversible permeability on cell membranes and increases the drug into the tumor [57,58]. Hyperthermia is a common strategy used in cancer therapy, where tumors are heated to temperatures slightly higher than normal body temperature and nanoparticles release their therapeutic agents in tumors [59].

## 7. SLNs as a Promising Alternative for Treating TNBC

SLNs were introduced as an alternative to other nanosystems, such as liposomes, micelles, and polymeric nanoparticles. SLNs have seen increased attention in recent years due to better biocompatibility and reduced systemic toxicity, avoiding the use of organic solvents [41]. Also, SLN-loaded drugs have shown a sustained release feature due to the use of solid lipids, providing a controlled drug release [60]. On the other hand, the stability of NSLs has also been studied. Soares et al. demonstrated that SLNs, freeze-dried even without cryoprotectant after six months and stored at 4 °C, showed an identical degree of initial morphology. Therefore, SLNs demonstrated retained therapeutic agents and structure after production and freeze-drying [61].

In addition, SLNs are an excellent drug delivery system since, as described above, they offer several characteristics, such as good bioavailability, good biocompatibility, controlled release kinetics, good encapsulation efficiency, and cost-effectiveness. This potentially makes SLNs a good choice over other formulations [39,62,63,64]. In addition, it has been observed that SLNs have shown extraordinary potential for clinical application.

On the other hand, SLNs have demonstrated higher loading capacity, improved entrapment efficiency, and reduced toxicity compared to micelles and liposomes. Dattani et al. developed three different paclitaxel-loaded nanoparticles (micelles, liposomes, and SLNs) to determine their potential clinical use. The results showed that paclitaxel-SLN has the slowest drug release rate and highest stability in human serum compared to micelles and liposomes; also, the in vivo studies demonstrated a decreased tumor growth rate compared with the other nanosystems. They concluded that paclitaxel-SLN is an effective system for target drug delivery [65].

Several studies are investigating using SLNs to enhance the therapeutic effect and overcome high proliferation rates and drug resistance. During the last few years, SLNs have been elaborated on with the encapsulation of different compounds, and these have been evaluated on TNBC cells. According to Paiva et al. (2022), SLNs loaded with docetaxel enhance the cytotoxic effect of the drug on MDA-MB-231 cells, a TNBC cell line [66]. This finding highlights the nanocarrier capability of SLNs, which are able to maintain and increase the antineoplastic activity of several drugs.

Chemoresistance during the TNBC treatment is a frequent event, and it has been associated with the presence of drug efflux pumps in tumor cells. One of the primary drug efflux pumps related to the development of acquired resistance is P-glycoprotein (P-gp/ABCB1) [67], so its inhibition has become a research focus. It has been reported that curcumin is an inhibitor of P-gp [68]; however, it is limited by low solubility and poor bioavailability [69]. One of the tools used to improve these physicochemical characteristics is encapsulating curcumin in SLNs. Fathy et al. reported that combining curcumin-SLN and doxorubicin effectively overcomes P-gp-mediated chemoresistance. Their study demonstrated that the formulation of curcumin-SLN increased two-fold more the intracellular accumulation of doxorubicin in the MDA-MB-231 cell line; this enhanced accumulation of doxorubicin is attributed to reduced P-gp mRNA because curcumin decreases intracellular ROS and causes consequent down-regulation of the Akt/IKK-α/β/NF-kB pathway [70].

Recently, quercetin has been tested as a treatment for breast cancer. In diverse studies, quercetin has exhibited low availability to tumor cells. One of the advantages of encapsulating quercetin in SLNs is the consequent increase in sustained release, thus improving the bioavailability of the compound in tumor cells. Hatami et al. reported that the encapsulation of quercetin in SLNs triggered a higher rate of apoptosis in an MDA-MD-231 cell line by regulating the expression of Bax and Bcl-2 genes [71].

The new generations of nanoparticles developed in the last few decades have allowed for the co-encapsulation of drugs with distinct types of molecules, such as peptides with genetic material. The addition of peptides or ligands to nanoparticles is reported to potentiate the targeting of tumor cells, achieving a greater accumulation of the encapsulated drug in the therapeutic target.

TNBC cells and tumor neovascular endothelial cells are known to overexpress various types of receptors, among which are those of α(v)β(3) integrins. The latter receptors play an important role in tumor invasion and metastasis. Several studies on the arginine-glycine-aspartate peptide (RGD) have evidenced its competitive binding to α(v)β(3) integrins, showing lower expression. Shan et al. demonstrated that the functionalization of SLNs with the RGD peptide increased the concentration of nanoparticles in tumor cells, leading to the inhibition of MDA-MB-231 cell adhesion and invasion [72].

**Figure 8:**
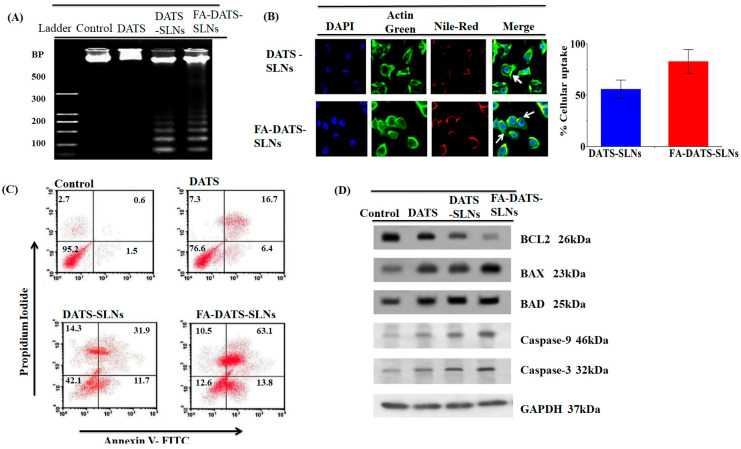
(**A**) DNA fragmentation of the MDA-MB-231 cell line treated with DATS, DATS-SLNs, and FA-DATS-SLNs. (**B**) Cellular internalization of DATS-SLNs and FA-DATS-SLNs in the MDA-MB-231 cells. (**C**) Apoptosis in MDA-MB-231 cells treated with DATS, DATS-SLNs, and FA-DATS-SLNs. (**D**) Blots of Bcl2, Bax, and Caspases 3, 9 in the MDA-MB-231 cells treated with FA-DATS-SLNs, DATS-SLNs, and DATS. Image from De, A et al. [54].

Due to the low therapeutic response observed in TNBC, different alternatives are being investigated, including natural compounds such as DATS (diallyl trisulfide), an antioxidant derived from garlic. It has been reported that DATS has a high cytotoxic effect on the MDA-MB-231 cell line [73]; however, this compound has limited bioavailability and a short half-life. Recently, De et al. reported a cytotoxic effect of DATS in an SLN (DATS-SLN) on the MDA-MB-231 cell line. Nevertheless, when DATS-SLN was functionalized with folic acid (FA) to target TNBC cells, a more significant cytotoxic effect was observed compared to DATS-SLN. FA-DATS-SLNs significantly reduced Bcl-2 and increased caspase 9 expression, thereby enhancing apoptosis [54]. The authors demonstrated that directing an SLN with FA improved the efficacy of DATS, paving the way for the development of targeted therapies with natural compounds (Figure 8).

**Figure 9:**
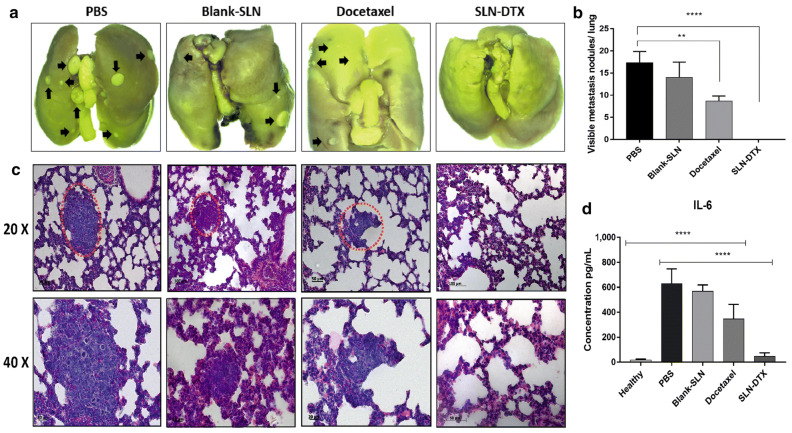
Lung metastasis prevention of SLN-DTX. (**a**) Lung images of different groups. (**b**) Numbers of tumor nodules on the lungs with different groups. (**c**) Histopathological images of the lungs with the treatments. (**d**) IL-6 serum levels of mice. The metastasis is shown in black arrows and red dotted circles. Data represent mean values  ±  standard error (** *p*  <  0.01; **** *p*  <  0.0001). Image from da Rocha, et al. [74].

Metastasis is the main cause of death in patients with TNBC. Although ongoing research has been carried out for many years on different strategies for improving the prevention and treatment of metastasis in cancer patients, conventional therapeutic methods have not produced a significant decrease in metastasis or the survival rate (22%) 5 years after the onset of metastasis. According to Da Rocha et al., the antitumor efficiency of docetaxel was improved by encapsulating it in SLNs, resulting in reduced spontaneous pulmonary metastasis in an orthotopic model of breast cancer (Figure 9) [74].

Functionalizing SLNs with molecules overexpressed in tumor cells and co-encapsulating different drugs to achieve a better therapeutic response is a strategy investigated in recent years. Chaudhuri et al. developed docetaxel- and erlotinib-loaded SLNs, which further surface functionalized by folic acid for increased tumor targeting to improve their anticancer activity against TNBC. The nanosystem (FA-DOC/ERL-SLNs) showed enhanced anticancer activity and decreased the migration capacity of MDA-MB 231 cells [75]. Over the last few years, research on SLNs for the treatment of TNBC has been increasing; overall, the aforementioned findings support the use of SLNs as a promising alternative (Table 2).

## 8. SLNs as a System for the Release of Nucleic Acids

Nucleic acids, such as small interfering RNA (siRNA) and microRNA (miRNA), have recently been applied to the treatment of diverse diseases. When administered with conventional approaches, they have a short half-life and are quickly eliminated from systemic circulation. However, genetic material encapsulated and transported in SLNs has displayed increased circulation time.

As one example of the versatility of SLNs, they have been synthesized with a net positive charge on their surface. These cationic SLNs (cSLNs) have been achieved by utilizing surfactant agents or ionic lipids with positively charged structural groups [81]. Among the lipids most commonly employed in such nanoformulations are didodecyldimethylammonium bromide (DDAB) and 1,2-dioleoyl-3-trimethylammonium-propane (DOTAP) [82].

cSLNs are one of the most efficient nanosystems for the transport of hydrophilic drugs and genetic material with low solubility in water. They hold promise for the targeted delivery of genes to treat a wide variety of diseases. Since the surfaces of cell membranes and serum proteins have a negative charge, they tend to interact better with cSLNs than other nanoparticles [81,83]. Yu et al. developed cSLNs loaded with paclitaxel and plasmid encoding an enhanced green fluorescent protein (pEGFP). This plasmid contains only the EGFP gene. The evaluation of their antineoplastic activity when using in vitro and in vivo models of breast cancer revealed an efficiency superior to that of conventional formulations. Furthermore, hyaluronic acid was added to the SLNs because the distinct pH of tumor cells reacts with it to stimulate the release of the drug and genetic material. Therefore, there was a greater accumulation in tumor tissue, leading to better biodistribution and reduced tumor size [84].

Liu et al. demonstrated that cSLNs are able to function as nanocarriers of miRNA, protecting it from degradation. They targeted breast cancer stem cells after co-encapsulating paclitaxel with miRNA 200C, observing an enhanced cytotoxic response compared to the same substances administered in conventional form [85]. Hence, cSLNs can protect nucleic acids from degradation and deliver them to their target.

Another strategy that has been recently investigated is the use of aptamers. Aptamers are single-stranded DNA or RNA sequences that recognize a specific target with high affinity [86]. Due to the fact that TNBC is highly heterogeneous, different therapeutic targets have been explored, including CD44 and the epidermal growth factor receptor (EGFR). CD44 is a transmembrane glycoprotein overexpressed in TNBC cells, playing a significant role in cell proliferation, migration, and invasion [87]. Thus, CD44 has become an essential therapeutic target in TNBC research. Darabi et al. developed and characterized an SLN with anti-CD44 and the epidermal growth factor receptor (EGFR) aptamers to release Doxorubicin. The targeted Doxorubicin delivery system showed a high cytotoxic effect on the MDA-MB-468 cell line compared to nanoparticles not decorated with anti-EGFR and CD44 [77]. Their results indicated that using aptamers in combination with chemotherapy in SLNs could be a promising strategy for TNBC treatment. Recently, there has been a Phase 1 clinical trial (NCT03739931) to evaluate the safety and tolerability of a lipid nanoparticle encapsulating mRNA-2752 (mRNA encoding OX40L T cell co-stimulator, IL-23, and IL-36γ pro-inflammatory cytokines) in advanced solid tumors and lymphoma; the authors reported that the nanoparticle with mRNA-2752 is safe and tolerable when combined with durvalumab. Enrollment is ongoing in expansion cohorts of TNBC, urothelial cancer, lymphoma, immune-checkpoint refractory melanoma, and NSCLC [88].

## 9. SLNs for the Delivery of CRISPR-Cas Systems

Several clustered regularly interspaced short palindromic repeat (CRISPR) screens have been conducted to identify genes associated with oncogenes and drug resistance in TNBC [89,90,91,92]. However, delivering the CRISPR systems requires appropriate vehicles. Lipid nanoparticles are commonly utilized as vehicles to deliver CRISPR due to their advantages like biodegradability, biocompatibility, and stability. The use of SLN-carrying CRISPR-Cas systems to treat TNBC represents a promising advance in gene therapy. SLN-carrying CRISPR-Cas systems enable precise edits to DNA, which may help eliminate mutations that maintain the development and progression of TNBC. Additionally, SNLs have the ability to improve the stability, bioavailability, and effectiveness of CRISPR-Cas delivery. Various studies are exploring the use of these combined technologies, and, to date, SLNs, as non-viral vectors, have been extensively researched for their ability to enable transfection in vivo and in vitro. However, there are few reports on CRISPR-Cas delivery SLN systems. Akbaba et al. developed a glycerol monostearate (GMS)-SLN system to encapsulate CRISPR-Cas9 in cancer treatment; they reported a monodispersed and stable system with a transfection efficiency of 38.5%. They concluded that SLNs are an excellent alternative as a CRISPR-Cas delivery system for genome editing-based applications [93]. Further research and development are expected to advance novel treatments with improved efficacy in TNBC.

## 10. Future Directions

Several preclinical and clinical studies have investigated using SLNs as a delivery nanosystem in breast cancer. SLNs offer several advantages: stability, low cost, and scalable production methods. Furthermore, SLNs can co-deliver multiple therapeutic agents, such as antineoplastic drugs, nucleic acids, or several target therapies. The future direction of SLNs in TNBC is promising; research efforts will likely focus on refining SLN formulations, optimizing targeting strategies for TNBC tumor cells to decrease side effects, and advancing their clinical translation to benefit patients.

## 11. Conclusions

SLNs are novel drug transport systems with advantages over other colloidal and polymeric nanocarriers. Their capacity to encapsulate therapeutic agents allows for a controlled and targeted release, thus improving the efficiency of cancer treatment and minimizing adverse effects. The co-encapsulation of drugs with genetic material has been achieved in some SLNs. Additionally, the surface of SLNs has been modified to enhance affinity for tumor cells. The ongoing development of SLNs as a therapeutic strategy opens new perspectives for treating TNBC. Although various studies have shown the great potential of SLNs as an alternative treatment for TNBC, further research is needed on the long-term safety and efficiency of these nanoparticles to be applied in clinical trials.

## Figures and Tables

**Table 1:** The main advantages and disadvantages of SLNs versus conventional systems of the administration of drugs [28,29,32].

Advantages	Capacity to incorporate and transport a wide variety of therapeutic agents. Greater physical and chemical stability. Capability of providing a controlled and sustained release of drugs. A decrease in toxicity and adverse effects of the encapsulated drugs. Improved biodistribution and bioavailability of the encapsulated drugs. Synthesis under conditions of low toxicity (in the absence of organic solvents). Simple and economical mass production. Biocompatible and biodegradable lipid systems. An increase in the solubility and stability of the incorporated molecules. Enhanced permeability of drugs through the blood–brain barrier. Better permeability of drugs throughout the organism and their retention in tissues. A broad spectrum of routes of administration, including oral, rectal, nasal, ocular, parenteral, and intravenous.
Disadvantages	Possibility of expulsion of the encapsulated drug during the storage period. An increase in the size of the particles during storage. Low charge efficiency of some hydrophilic drugs. The accumulation of lipids in the liver and spleen can cause pathological alterations. Potential polymorphism transitions.

**Table 2:** List of SLN pre-clinical trials for TNBC.

Nanoparticle	Composition	Drug	Method Used	Phase	Results	Reference
Folic acid (FA)-conjugated SLN FA-DOC/ERL-SLN	Lipid: Precirol ATO 5 Surfactant: Tween 20	Docetaxel and erlotinib	High shear homogenization	In vitro	FA-DOC/ERL-SLN enhanced anticancer activity and further inhibited the colony-forming ability and the migration capacity of MDA-MB-231 and 4T1 TNBC cells.	[75]
QC-SLN	Lipid: Compritol 888 ATO Surfactant: Oleic acid and lecithin	Quercetin	Hot homogenization	In vitro	QC-SLN decreased cell viability, colony formation, and angiogenesis and increased apoptosis in MDA-MB-231.	[71]
Folic acid (FA)-conjugated SLN FA-DATS-SLN	Lipid: Palmitic acid Surfactant: Pluronic F-68 Co-surfactant: Soy lecithin	DATS (diallyl trisulfide)	Hot homogenization	In vitro	FA-DATS-SLNs downregulate anti-apoptotic proteins (Bcl2) and upregulate pro-apoptotic caspase-9 in MDA-MB-231	[54]
CS/Lf/PTS-SLN	Lipid: Compritol^®^ 888 Surfactant: P407	Chondroitin/Lactoferrin-dual functionalized pterostilbene	Ultrasonication	In vitro, in vivo	In vitro studies on triple-negative MDA-MB-231 have shown significant cytotoxicity of CS/Lf/PTS-SLNs. The in vivo anti-tumor efficacy of CS/Lf/PTS-SLNs decreased tumor growth compared to the PTS solution.	[76]
SLNs/DOX/Dexa/CD44/EGFR aptamers	Lipid: glycerol monostearate (GMS) Surfactants: Soy lecithin and Tween 80	Doxorubicin, anti-EGFR/CD44 dual-RNA aptamers, and Dexamethasone_LHON	Double emulsification (W1/O/W2) and solvent evaporation	In vitro	SLNs loaded with doxorubicin and decorated with CD44 and EGFR aptamers significantly inhibited cell proliferation compared to SLNs without aptamers in the MDA-MB-468 cell line.	[77]
CURC-SLN	Lipid: Cholesterol/stearoyl chitosan Surfactant: Epikuron^®^200 Co-surfactant: Cremophor^®^RH60	Curcumin	Cold dilution of microemulsion	In vitro	CURC-SLN and doxorubicin effectively overcome P-gp-mediated chemoresistance. CURC-SLN increased 2-fold more the intracellular accumulation of doxorubicin in the MDA-MB-231 cell line.	[70]
PBA-Niclo-SLN	Lipids: Phenylboronic acid, stearylamine (PBSA) Surfactant: Tween 80 and pluronic F68	Niclosamide	Emulsification-solvent evaporation	In vitro, in vivo	The PBA-Niclo-SLN showed increased cytotoxicity, apoptosis, and arresting cell cycle in triple-negative breast cancer (MDA-MB-231). The PBA-Niclo-SLN decreased tumor growth in TNBC tumor-bearing female C57BL/6J mice.	[78]
SLN-DTX	Lipid: Compritol Surfactant: Span 80 and Pluronic F127	Docetaxel	High energy	In vitro	SLN-DTX enhance the cytotoxic effect of the drug on MDA-MB-231.	[66]
sFA-SLNs-LTZ	Lipid: Glycerol monostearate powder (GMS) Surfactant: Poloxamer 188	Letrozole	High-pressure cold homogenization	In vitro, in vivo	LTZ-SLNs showed significant cytotoxic properties in MDA-MB-231. LTZ-SLNs minimize systemic toxicity.	[79]
SLN-DTX	Lipid: Compritol Surfactant: Span 80 and Pluronic F127	Docetaxel	High energy	In vivo	SLN-DTX significantly reduced tumor volume compared to free docetaxel and prevented lung metastasis in 4T1 tumor.	[74]
Res-SLN	Lipid: Stearic acid Surfactant: Soy lecithin and Tween^®^ 80	Resveratrol	Solvent injection	In vitro	Decreased cell viability in MDA-MB-231 cell line. Decreased the expression of cyclinD1 and c-Myc.	[80]

## Data Availability

The data that support the findings of this study are available on request to the corresponding author.
